# Heterogeneity Matters: Predicting Self-Esteem in Online Interventions Based on Ecological Momentary Assessment Data

**DOI:** 10.1155/2019/3481624

**Published:** 2019-01-13

**Authors:** Vincent Bremer, Burkhardt Funk, Heleen Riper

**Affiliations:** ^1^Institute of Information Systems, Leuphana University, Lueneburg, Germany; ^2^Department of Clinical, Neuro- & Developmental Psychology, Vrije University, Amsterdam, Netherlands; ^3^Amsterdam Department of Health Sciences, Vrije University, Amsterdam, Netherlands

## Abstract

Self-esteem is a crucial factor for an individual's well-being and mental health. Low self-esteem is associated with depression and anxiety. Data about self-esteem is oftentimes collected in Internet-based interventions through Ecological Momentary Assessments and is usually provided on an ordinal scale. We applied models for ordinal outcomes in order to predict the self-esteem of 130 patients based on diary data of an online depression treatment and thereby illustrated a path of how to analyze EMA data in Internet-based interventions. Specifically, we analyzed the relationship between mood, worries, sleep, enjoyed activities, social contact, and the self-esteem of patients. We explored several ordinal models with varying degrees of heterogeneity and estimated them using Bayesian statistics. Thereby, we demonstrated how accounting for patient-heterogeneity influences the prediction performance of self-esteem. Our results show that models that allow for more heterogeneity performed better regarding various performance measures. We also found that higher mood levels and enjoyed activities are associated with higher self-esteem. Sleep, social contact, and worries were significant predictors for only some individuals. Patient-individual parameters enable us to better understand the relationships between the variables on a patient-individual level. The analysis of relationships between self-esteem and other psychological factors on an individual level can therefore lead to valuable information for therapists and practitioners.

## 1. Introduction

Access to mental care is limited; by providing further access, Internet-based interventions can close the gap between treatment and demand [1–3]. At the same time, online-based interventions may lead to comparable outcomes compared to face-to-face treatment [1, 4]. In Internet-based interventions, data about various psychological factors, for example, the self-esteem level of individuals, is often collected. Self-esteem is closely related to psychological well-being and satisfaction with life [5]. Low levels of self-esteem are associated with serious mental problems such as depression, anxiety [6], or eating disorders [7]. Trzesniewski et al. [8] found that low self-esteem can lead to “negative real-world consequences” such as mental and physical health problems, misconduct, and worse economic outlooks. In the literature, however, there is a debate if low mood levels affect self-esteem or vice versa. Two models exist for each assumption. The vulnerability model assumes that self-esteem is a risk for depression whereas the scar model interprets self-esteem rather as an outcome or aftermath of depression [9]. One study, for example, found that low self-esteem can predict depression decades later [10]. Steiger et al. [11] found that the vulnerability and the scar model are valid over decades with weaker effects for the scar model. A reoccurring finding is that low levels of self-esteem are associated with serious mental illnesses which in turn are known to be associated with decreased quality of life and tremendous health care costs, as well as increased costs for individuals and governments [5–7, 12–14]. Thus, we aimed at predicting the self-esteem level of individuals in this study and analyze its relationships with a variety of psychological factors.

Data about self-esteem and other psychological factors such as mood levels or social interactions are often assessed by Ecological Momentary Assessments (EMA). These EMA methods collect data regarding behavior, symptoms, and cognition close in time to the users' experience and in their natural environment [15, 16]. Diaries, which are used for the analysis in this paper, are one example of EMA methods that are often utilized [15].

Due to multiple measures per individual, this data has a nested structure [17, 18]. As is common in the social sciences [19], self-reports of diary data can be ranked on an ordinal scale. Individuals are often prompted to rank their mood level, for instance, by providing a score between one and ten for a specific question such as “*How is your mood right now?” *Data with this structure needs to be analyzed by utilizing appropriate statistical models that can account for the ordinality in the measurements, for example, ordinal logit models or generalized linear models. In research studies, however, this is often not the case [20–22]. Jakobsson [20] and LaValley and Felson [21] analyzed a multitude of journal articles; eventhough ordinal scales were often used, they came to the conclusion that frequently there were no appropriate data representation techniques or data analysis methods present. They found that solely 49% (La Valley et al.: 39.4%) of the analyzed articles had proper data presentation and 57% (La Valley et al.: 63.4%) had appropriate data analysis. This is alarming since an improper handling can lead to bias and incorrect interpretation of statistical effects [23].

Each patient behaves differently, has different experiences, and can be affected by psychological factors in various ways. Repeated measurements provided by patients can therefore not be considered to be independent [24]. Considering the differences among patients by implementing patient-individual parameters might lead to a better model fit (representation of the pattern in the data) and an increased prediction performance (ability to predict unobserved values of the dependent variable). By revealing these patient-individual parameters, individual effects for the independent variables (psychological factors) can be obtained for each patient, which in turn can result in individualized decision support systems and subsequently individualized recommendations in a clinical context.

In this study, we thus combined ordinal models appropriate for the analysis of diary data, namely, the ordinal logit model [25, 26] and the less frequently utilized stereotype logit model [26, 27], and proposed to extend the models by including patient-specific parameters in order to account for heterogeneity among the participants. General mixed models are often applied when analyzing data that includes repeated measurements [24]. Hedeker [23], for example, discussed mixed effects logistic regression models for ordinal data and illustrated a possible hierarchical structure in which the effect each patient has on the outcome value is considered. In contrast to this study, our approach considered different influences of the psychological factors on the individuals which led to individual slopes. These patient-specific coefficients can potentially result in more information on how the analyzed psychological factors are related to the self-esteem of the patients on an individual level and can therefore lead to a knowledge gain for researchers and practitioners. We applied the models to self-reported diary data from an Internet-based depression treatment [28] in order to predict the self-esteem of individuals. At the same time, we revealed the relationship between a variety of psychological/psychosocial factors (mood, worries, sleep, enjoyed activities, and social contact) and the self-esteem level of patients. Thus, this study contributes to existing research by gaining insight into the patients' behavior and how their self-esteem is related to a variety of factors on an individual level and thus by highlighting the importance of individuality in this context.

## 2. Materials and Methods

### 2.1. Data

The data we utilized for our approach is acquired from an EU funded two-arm randomized control trial that compared bCBT (blended cognitive behavior therapy, experiment group) and face-to-face treatment (control group) [28]. Participants were 18 years or older, met criteria for a major depressive disorder, were not of high suicidal risk, were not currently being treated for depression, and had access to an Internet connection. The utilized data was based on diary data that has been assessed in the study through an EMA mobile-application between February 2015 and January 2017. The diary questions were sent via email or text message depending on the therapists' choice. The mood level of the participants was collected every day at a random time between 10 a.m. and 8 p.m. All other factors were collected on specific days; the first and last seven days of the intervention and one random day each week in the intervention period. All factors could be ranked on a scale from one to ten. We only utilized days on which all factors were assessed, which resulted in the analysis of 130 patients and their 2326 observations including all psychological factors that will be introduced in the following.

**Self-Esteem **| The dependent variable in our analysis was the self-esteem of the patients. It was assessed through the question “How do you feel about yourself right now?” This question is closely related to an item of the state self-esteem scale [29] and can represent a person's self-image [30]. The same question has also been utilized in another study that measured self-esteem for individuals and has shown to be correlated with the Rosenberg self-esteem scale [31–33]. In this study, we defined this question as the self-esteem level.

**Mood **| Mood is an important factor for an individual's well-being, physical health, and behavioral patterns [34, 35]. We analyzed the relationship between these factors and hypothesized that the mood level is positively related to self-esteem. This predictor was assessed by the question “How is your mood right now?”

**Worry **| Worries are connected to anxiety disorders [36] and depression [37]. Since the act of worrying can potentially create feelings and thoughts that impact self-respect or cause individuals to underestimate themselves, it could be linked to self-esteem. We hypothesized that this factor is negatively related to the self-esteem of the patients. Worries were assessed by asking the patients “How much do you worry at the moment?”

**Sleep **| Sleep supports various functions of the human body such as repair and restorative processes [38] and is a crucial aspect for the well-being of an individual [39]. Prior research found that low levels of sleep can lead to lower self-esteem [40]. We hypothesized that “good” self-reported sleep levels can lead to higher levels of self-esteem. Sleep was assessed through the question “How well did you sleep last night?”

**Enjoyed Activities **| This concept relates to any action that has been executed by the participant that day. It describes to what degree the patient has relished a specific day by the performed activities. Since we assumed that joy—that in turn can trigger happiness—can potentially boost the self-esteem of individuals, we hypothesized that enjoyed activities are positively linked to self-esteem. The predictor enjoyed activities was assessed by the question “How much did you enjoy activities today?”

**Social Contact **| Social contact can provide important emotional support; and the lack thereof can be linked to depression [41]. We hypothesized a positive relationship between social contact and self-esteem. Social contact was assessed by asking the individuals “How much were you involved in social interaction today?”

### 2.2. Statistical Analysis

#### 2.2.1. Approach

**Figure 1:**
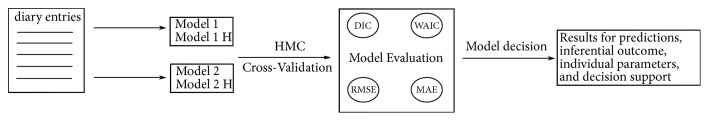
Graphic visualization of approach.

We applied two different models for predicting the self-esteem at time* t* based on the aforementioned predictors and their scores at time* t*, the ordered logit and stereotype logit model. Both approaches account for the ordinality in the measurements. Four models were eventually used because we modified each method by implementing patient-specific parameters in order to consider how they are individually affected by the psychological factors (Figure 1). We used Hamiltonian Monte Carlo techniques (HMC) for parameter estimation [42], applied cross-validation, and evaluated the models by comparing their outcomes based on various performance measures. We then utilized the model that performed best for illustrating the concrete predictions, the inferential outcomes (relationship between psychological factors and self-esteem), and the patient-individual parameters.

#### 2.2.2. Ordinal Logistic Regression Model

One method that was utilized is the frequently used proportional odds or ordered logit model (OLM) that was initially proposed by McCullagh [25]. This model estimates the odds of observing a specific rank or less of self-esteem (score on the scale) for patient* j* at time step* t* for *rank* = 1,…, *C* where *C* is the number of ranks or the highest category on a scale (ten in our analysis since self-esteem is rated on a scale from one to ten) [43]:

(1) $$\begin{array}{c} \theta_{cjt}=\frac{P\left(rank\leq c\;|\;x_{jt}\right)}{P\left(rank>c\;|\;x_{jt}\right)}. \end{array}$$

The estimation then follows (2). The parameters *α*_*c*_ are the boundaries of the categories or thresholds, also called cutpoints where *c* = 1,…, *C* − 1. This parameter has therefore nine distinct values. Furthermore, the cutpoints are following the constraint *α*_1_ ≤ *α*_2_ ≤ ⋯ ≤ *α*_*C*−1_. *x*_*jt*_ is a vector of length five that represents the observations of the psychological factors for each patient* j* at each time step* t*. The *β* parameters are the weights to be estimated that reveal relationships between the factors and are utilized for the self-esteem prediction. This model is based on the proportional odds assumption. This means that the OLM assumes all *β* terms and their effects to be equal among all the levels of the dependent variable. As we can see, the *β* parameters do not vary among the ordinal levels or in any other fashion. Therefore, no individual effects are captured. The fixed *β* coefficient for all patients in the data leads to the unrealistic assumption that all individuals are similarly related to the psychological factors.

(2) $$\begin{array}{c} \ln\left(\;\theta_{cjt}\;\right)=\alpha_{c}-\left(\;x_{jt}\beta \;\right) \end{array}$$

However, humans possess very unique and intricate qualities; each person has a different personality, opinion, thinking structure, and behavior; this can in turn lead to patient-individual effects from the predictors [44, 45]. We further assumed that including patient-individual parameters could lead to a greater prediction performance because more variance can potentially be explained. However, this process comes with a sacrifice of an increased model complexity. Nevertheless, we modified the model by introducing an additional index* j* into the *β* parameters which accounts for the varying effect a predictor can have on an individual. The OLM then yields the following form:

(3) $$\begin{array}{c} \ln\left(\;\theta_{cjt}\;\right)=\alpha_{c}-\left(\;x_{jt}\beta_{j}\;\right). \end{array}$$

#### 2.2.3. Stereotype Ordinal Logit Model

Another model that is less frequently used in research, presumably due to the rare existence of already implemented software packages [26, 46], is the stereotype ordinal logit model. This model was created by Anderson [27] in order to tackle the restrictive nature of the OLM due to its proportional odds assumption that is often violated in real datasets [47]. It can be seen as an extension of the multinomial logistic regression with the distinction that less parameters have to be estimated [46]. We additionally applied this model in order to compare the performance of both techniques and to demonstrate that heterogeneous parameters are not only beneficial when utilizing the OLM, but also in other statistical procedures. As in the OLM, *θ*_*cjt*_ is estimated; this is the odds of observing a specific rank of self-esteem in comparison to a baseline category (in our case the last category ten) for patient* j* at time* t*.

(4) $$\begin{array}{c} \theta_{cjt}=\frac{P\left(rank=c\;|\;x_{jt}\right)}{P\left(rank=C\;|\;x_{jt}\right)} \end{array}$$

The procedure of the stereotype logit model for the estimation is illustrated in (5) for *c* = 1,…, *C*. As we can see by the index* j*, the *β* parameters already consider individual effects. The original model does not include this index. The *α*_*c*_′*s* are the intercepts and the *ϕ*_*c*_ parameters are a score for the different levels of the outcome variable where *α*_1_ = *ϕ*_1_ = 0 [46]. Ordinality is only given as long as the constraint 0 = *ϕ*_1_ ≤ *ϕ*_2_ ≤ ⋯≤*ϕ*_*C*_ = 1 is considered. Specifically, for a four-point scale, two *ϕ*′*s* are to be estimated. For a ten-point scale, eight *ϕ*′*s* are to be estimated.

(5) $$\begin{array}{c} P\left(\;rank=c\;|\;x_{jt}\;\right)=\frac{\exp\left(\alpha_{c}+\phi_{c}x_{jt}\beta_{j}\right)}{\sum_{c=1}^{C}\exp\left(\alpha_{c}+\phi_{c}x_{jt}\beta_{j}\right)} \end{array}$$

#### 2.2.4. Parameter Setting

**Figure 2:**
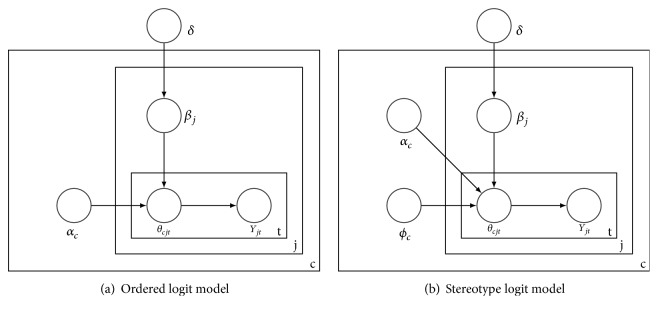
Graphic visualization for both models as plate notation.

Enabled by the Bayesian approach, we set different priors based on assumptions and already existing literature mentioned above. In this context, priors are beliefs in terms of probability distributions about the effects of the predictors that can be set before the actual data is considered. We set weak positive priors for the predictors mood, sleep, enjoyed activities, and social contact. For the variable worry, we set a weak negative prior. Implementing weak priors means sampling the corresponding parameter with high variance. Thereby, prior knowledge from related literature is taken into account while at the same time, the data strongly affects the analyses.  Figure 2 illustrates the hierarchical structure of both models including heterogeneity parameters as a plate notation.

The parameters are distributed as shown in (6) where *σ*^2^ = 100 (high variance). The expected value for the hyperparameter *δ* is -1 or 1 depending on the definition as either a weak negative or positive prior. The parameters *δ* and *α*_1…*C*_ are sampled from a normal distribution. The heterogeneous parameters for each patient, *β*_1…*J*_, are also sampled from a normal distribution; however, they are based on the vector *δ*. We decided to sample from a normal distribution because this allowed the parameters to evenly take on a positive or negative value. This means that we assumed that patients exist for whom a specific coefficient is positive whereas other patients are negatively affected. The results for *δ* indicate the effects each predictor has on the self-esteem on a population level. We utilized this parameter for prediction for the models that do not consider heterogeneity. The *β* parameters for each patient were used for the prediction of the individual models and illustration of the individual parameters.

(6) δ~Nμ∈−1,1,σ2αc~N0,σ2γc~DirAβj~Nδ,σ2Yjt~Catθcjt

Solely in the stereotype model, as we can see in Figure 2, *θ*_*cjt*_ also depends on *ϕ*_*c*_. This parameter is the cumulative sum of *γ*_*c*_ which follows a *Diric*h*let*(*A*_1_,…, *A*_*C*_) distribution where *A*_1…*C*_ = 1. Since the stereotype model requires *ϕ*_*c*_ to be steadily increasing, initialized with 0 and be limited to 1, sampling *γ*_*c*_ from a Dirichlet distribution is an appropriate procedure to meet this constraint [46]. As a final step, the actual predicted self-esteem level for each individual at each point in time (*Y*_*jt*_) is sampled from a categorical distribution based on *θ*_*cjt*_. For each model, we performed 60,000 iterations on four chains when running the Hamiltonian Monte Carlo algorithm and stored every twentieth draw from the last 30,000 iterations. We implemented the models in Python (https://www.python.org/) and utilized STAN [42] for Monte Carlo procedures.

#### 2.2.5. Performance Measures

We implemented 10-fold stratified cross-validation in order to determine the model that achieves the best prediction performance. In 10-fold cross-validation, the dataset is divided into ten equally sized chunks (in our case each patient has observations in the training as well as the test dataset). Then, the models are trained on nine chunks and the tenth is predicted. This process is repeated ten times until every chunk is utilized as test data. 10-fold cross-validation is widely used and has also been shown to be suited for real-world datasets [48, 49].

We utilized the Deviance Information Criterion (DIC) [50] as indicator for measure of fit and model complexity [51]. The DIC is often used for model comparison and selection, especially in a Bayesian context [52]. The performance of a model is evaluated by the trade-off between how well the model fits the data and the complexity of the model. The model fit is expressed by the deviance (the lower the value, the better the fit), which is essentially the difference between a saturated model (a model that explains all variance in the responses) and the actual model. A penalty term is added to the model fit that is increasing with a rise in number of parameters [50]. Thus, models are preferred that have a smaller number of parameters. We chose the DIC as an indicator for model selection and comparison because it has been performing sufficiently regarding a variety of examples [51, 53].

According to Ando [54] and Richards and Richardson [55], however, the DIC can tend to prefer overfitted models and is only based on a point estimate [56, 57]. Thus, we also utilized the widely applicable or Watanabe-Akaike information criterion (WAIC) [58]. The WAIC is infrequently used in research and practice because of its additional computational effort [57]. According to Vehtari et al. [57], the WAIC represents an improvement of the DIC. Since the calculation for the number of parameters is based on each data point of the log likelihood, which is not the case for the DIC, the outcome is more stable and reliable. The WAIC (as well as the DIC) suggests a superior performance the smaller the value. For reasons of comparison and because of the mentioned issues regarding the DIC, we utilized both measures in our analyses. For readers interested in the exact derivations and steps regarding the calculation of the DIC and WAIC, we refer to the papers of Spiegelhalter et al. [50] and Vehtari et al. [57], respectively.

We further used the root-mean-square error (RMSE) and mean absolute error (MAE) as performance indicators. There is a debate about the selection of choosing either one of these measures. Willmott and Matsuura [59] and Willmott et al. [60], for example, criticized the usage of the RMSE and came to the conclusion that it is not a good indicator for the average model performance. They emphasized to only utilize the MAE since it is more natural compared to the RMSE. However, Chai and Draxler [61] showed that the RMSE can be a better indicator for model performance. Since there is no specific agreement in the literature as to which measure is more reliable, we decided to report both measures in our analysis.

Additionally, we defined a* mean model*. This model uses the arithmetic mean of the self-esteem value among the whole training set as prediction for each self-esteem value in the test data. Since we included heterogeneous parameters, we also used a* mean individual model* that utilizes the arithmetic mean of the training set on an individual patient level as predictions. We used these measures for comparison and as a baseline model; if we would not achieve a higher prediction performance than the* mean models*, it is questionable if the creation of such complex models is even worth the effort.

## 3. Results and Discussion

### 3.1. Principal Results

We can see that the* mean individual model* clearly performed better compared to the* mean model* (Table 1). It is also indicated that all created models performed better than the* mean models* regarding the RMSE and MAE (the other performance measures are not generatable for the* mean models*). Indicated by a Wilcoxon-Test, the errors differed significantly (*P* < .05). Therefore, creating such models is beneficial in regard to predictive performance in this context. The results further indicate that the implementation of patient-individual parameters was advantageous; both models performed better regarding each of the performance measures when accounting for individual effects even though the complexity of the models (number of parameters) increased (indicated by DIC as well as WAIC). This result highlights the importance of accounting for individual parameters. We can further see that the stereotype logit model benefit more from heterogeneity. Thus, we decided to utilize this model for further demonstration and analysis.

**Figure 3:**
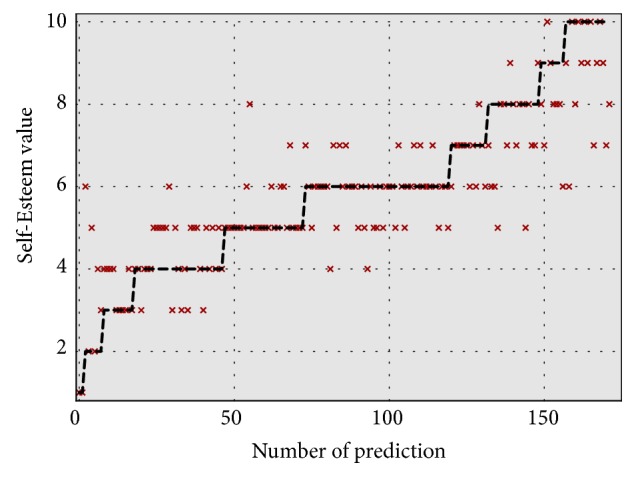
Graphic visualization of predicted and observed values.

Figure 3 illustrates the predictive performance of this model in more detail. Specifically, it shows the observed values of self-esteem in the test data as a line and the predictions of the test data as crosses. The values are sorted in ascending order according to the observed values. Oftentimes, the predictions were the exact observed self-esteem value. Only once, the prediction was four categories off; however, it was frequently falsely predicted with a distance of two ranks. Since the predictions were close to the observed value most of the time, also indicated by the performance measures, we consider this a good result.

Table 2 demonstrates the effects of the psychological factors on the self-esteem. Here, the analysis was executed based on all data without withholding observations for evaluation of the models. The results indicate that the mood level of the patients is significantly related to the self-esteem.

Since recent literature found that low self-esteem is linked to depressive moods [62] and mood changes can modify self-concepts [63], this finding is plausible. As already indicated by Scheier et al. [64], who found that enjoyable leisure activities are related to factors for well-being, we show that enjoyed activities significantly increased the self-esteem. When individuals experience certain activities as fun and pleasure, they might be involved in actions that can boost their confidence, be of avail, and foster feelings of happiness that can in turn increase the sense of self-worth. Therefore, joy and doing well in a specific activity can potentially lead to feelings of reward and satisfaction and thus to an increased self-esteem.

**Figure 4:**
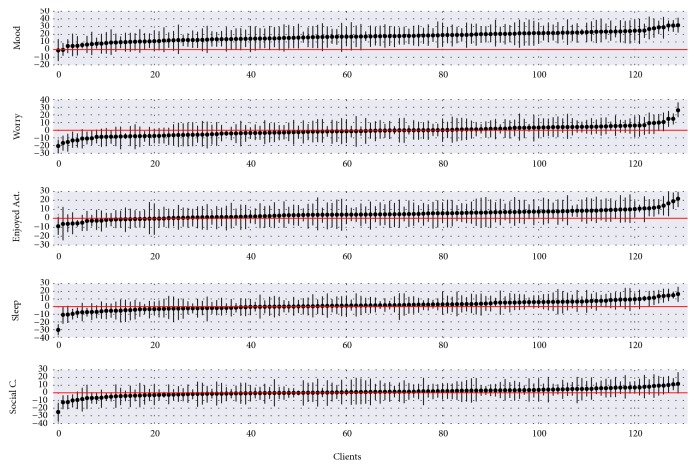
Graphic visualization of parameter distribution for each patient.

The other predictors were not significant. However, for some of the patients, these predictors might be significantly related to the self-esteem.  Figure 4 illustrates the distributions of the individual *β* parameters for each patient and each predictor. The values in this Figure cannot be read horizontally for each patient among the predictors; this means that the first patient for one predictor is not the same as the first patient for another predictor because the values are sorted in ascending order according to the individual mean value of the corresponding distribution. The horizontal line represents the zero value for the parameter and is an indicator for significance. The parameters varied tremendously, which again indicates the importance of considering heterogeneity. Even though the overall result for the variable worry, for instance, was insignificant, individuals exist for whom the outcome, the negative effect, was significantly true and vice versa. This finding occurs for every predictor except the mood level. Mood seemed to not be negatively related to self-esteem for any patient. Thus, the overall parameter for this predictor was highly significant. This individualized information can potentially help therapists to make refined and improved decisions on an individual level. Some patients were affected negatively by certain factors and some positively; with this procedure, it is possible to detect those specific patients. The gained information can lead to an increased understanding of patient-individual behavior and improved decision-making which can in turn result in personalized interventions and potentially better treatment outcomes.

### 3.2. Limitations

Besides the implications this study provides, we also depict some limitations and directions for further improvement and research opportunities. One limitation is the usage of diary data. Self-reported data is not inspected personally by a professional; even though this fact enables researchers to collect data in their natural environment, it lacks objectivity and can also lead to falsely reported data and social desirability bias [16, 65]. Furthermore, we measured self-esteem only based on one question. Even though this question is related to one item of the state self-esteem scale [29], it might not represent the whole complexity of self-esteem. We also obtained data for only 130 patients and 2326 observations. We believe that applying the modified models on other datasets in order to confirm the results can lead to an increased representativity. More data could improve the accuracy of gained information and especially enhance prediction performance. Therefore, more research in this context is necessary for a verification of the results.

Another aspect that can be viewed critically is the attempt of predicting a self-esteem value of a* new* patient that has not been seen before by the model. Unfortunately, even though we would have access to varying parameters for the individuals, we would not have any information on the* new* patient; therefore, we would predict the new patients' self-esteem based on the overall parameter *δ*. In fact, we would not perform less accurate compared to models that do not account for heterogeneous influences; however, we would also not benefit from the modified models. Nevertheless, after obtaining some information about the* new* patient and a recalculation of the models, we could obtain individual parameters for this patient. Thus, the utilization of the modified models is initially not beneficial for* new* patients, but after an initial data collection period, valuable results can be generated.

Another important aspect is the question of the exact impact of more accurate predictions. How can the illustrated improvement be translated into practical benefits? If a therapist is able to provide more refined recommendations, how are the individuals affected, how can this be converted into higher outcomes, and what role do costs play in this question? We seek to tackle challenges in this context in further research.

## 4. Conclusion

In this study, we predicted the self-esteem level of participants based on collected EMA data from a two-arm randomized control trial. We modified two statistical models by including heterogeneous slopes for each patient and employed Hamiltonian Monte Carlo techniques for parameter estimation. Therefore, one purpose of this study was to highlight the importance of individuality in such analyses. We illustrated a path of how individual parameters can be considered in an ordinal context and demonstrated how the prediction performance of different models is influenced by doing so. Individual parameters did not only increase the performance of these models but also allow practitioners to investigate differences among patients; possibly leading to knowledge gain and deeper insight about the patients. We further emphasized the importance of self-esteem in this context and investigated its relationships with other psychological factors. We found that the self-esteem level of patients was positively related to mood and when individuals experienced joyful activities. We further found that worries can be negatively linked to self-esteem whereas better sleep and social contact can be positively related to self-esteem. These latter results were not significant overall; however, we demonstrated that for some individuals these effects are significant. With our approach, we hope we can provide valuable information in the mental health sphere and support the decision-making process in personalized interventions.

## Figures and Tables

**Table 1:** Results: performance for each model based on performance measures.

**Model**	**RMSE**	**MAE**	**DIC**	**WAIC**
No Heterogeneity Ordered logit	1.20	0.81	6204.88	6205.87

Heterogeneity Ordered logit	1.18	0.80	5914.91	6143.56

No Heterogeneity Stereo	1.21	0.82	6364.25	6369.72

Heterogeneity Stereo	1.08	0.73	5871.31	5772.14

Mean model	1.90	1.48	-	-

Mean individual model	1.38	0.98	-	-

**Table 2:** Results: estimated model parameters including High Density Interval (significant parameters in bold).

**Variables**	**Median**	**2.5**%** HDI**	**97.5**%** HDI**
Mood	**16.82**	**14.25**	**19.55**

Worry	-1.05	−2.95	0.69

Sleep	1.50	-0.52	3.42

Enjoyed Activities	**4.26**	**2.37**	**6.24**

Social Contact	0.81	-1.34	2.82

## Data Availability

The data used to support the findings of this study might be available from the corresponding author upon request.
